# A Step Towards Personalized Sports Nutrition: Carbohydrate Intake During Exercise

**DOI:** 10.1007/s40279-014-0148-z

**Published:** 2014-05-03

**Authors:** Asker Jeukendrup

**Affiliations:** 1 Gatorade Sports Science Institute, Barrington, IL USA; 2 School of Sport and Exercise Sciences, University of Birmingham, Edgbaston, B15 2TT UK

## Abstract

There have been significant changes in the understanding of the role of carbohydrates during endurance exercise in recent years, which allows for more specific and more personalized advice with regard to carbohydrate ingestion during exercise. The new proposed guidelines take into account the duration (and intensity) of exercise and advice is not restricted to the amount of carbohydrate; it also gives direction with respect to the type of carbohydrate. Studies have shown that during exercise lasting approximately 1 h in duration, a mouth rinse or small amounts of carbohydrate can result in a performance benefit. A single carbohydrate source can be oxidized at rates up to approximately 60 g/h and this is the recommendation for exercise that is more prolonged (2–3 h). For ultra-endurance events, the recommendation is higher at approximately 90 g/h. Carbohydrate ingested at such high ingestion rates must be a multiple transportable carbohydrates to allow high oxidation rates and prevent the accumulation of carbohydrate in the intestine. The source of the carbohydrate may be a liquid, semisolid, or solid, and the recommendations may need to be adjusted downward when the absolute exercise intensity is low and thus carbohydrate oxidation rates are also low. Carbohydrate intake advice is independent of body weight as well as training status. Therefore, although these guidelines apply to most athletes, they are highly dependent on the type and duration of activity. These new guidelines may replace the generic existing guidelines for carbohydrate intake during endurance exercise.

## Introduction

In the early 1900s, it was discovered that carbohydrate was an important fuel for exercise [1]. In 1939, a paper was published showing that carbohydrate use during exercise could be influenced by diet and that this could have an effect on exercise tolerance [2]. In the 1960s, it became clear that muscle glycogen played a significant role during exercise [3], and in the 1980s, the first studies showed that carbohydrate ingestion during exercise improved exercise capacity [4, 5]. No major advances were made in the next 20 years until about 2004, which marked the beginning of an era with a series of major breakthroughs with respect to carbohydrate feeding during exercise.

As these breakthroughs and their effects on sports nutrition became available over time, recommendations for athletes also evolved during this period. In more recent guidelines, it is generally accepted that carbohydrate intake is important to optimize endurance performance but recommendations are still not very specific [6]. Studies had demonstrated that relatively small amounts of carbohydrate (20 g/h) were sufficient to observe a performance benefit [7, 8]. Based on a study by Fielding et al. [7], it was believed that a minimum of 22 g of carbohydrate per hour was required to observe a performance benefit. Subjects exercised for 4 h and performed a sprint at the end. Performance improvements were observed when 22 g of carbohydrate was ingested every hour, whereas no effects were observed when half this dose was consumed (11 g/h). In a study by Maughan et al. [8], the intake of 16 g of glucose per hour improved endurance capacity by 14 % compared with water (no placebo was given in this study). At the same time, other studies suggested that exogenous carbohydrate oxidation never exceeded 60 g/h [9], and therefore this has often been used as an upper limit for carbohydrate intake during exercise. The most recent guidelines by the American College of Sports Medicine (ACSM) state that a carbohydrate intake of 30–60 g/h is recommended during exercise [6]. This is a relatively wide range and is independent of the type of activity, the duration of the activity, or the level of the athlete. With the evidence from studies and new insights obtained in the past 5–10 years, it is possible to provide more prescriptive and precise advice to athletes. It is beyond the scope of this review to discuss all the underlying evidence in great detail, as this has been done in several other recent reviews [10–15]. However, the purpose of this review is to consolidate the different pieces of carbohydrate intake information and translate our current understanding into practical guidelines for athletes competing in different events.

## Carbohydrate Ingestion During Exercise and Performance

Although the exact mechanisms are still not completely understood, it has been known for some time that carbohydrate ingestion during exercise can increase exercise capacity and improve exercise performance (for reviews see Jeukendrup [12, 15]). In general, during exercise longer than 2 h, carbohydrate feeding will prevent hypoglycemia, will maintain high rates of carbohydrate oxidation, and increase endurance capacity compared with placebo ingestion. It was initially believed that the duration of exercise had to be at least 2 h for carbohydrates to have an effect.

However, more recently, it has become clear that carbohydrate ingestion during exercise can improve exercise performance even during shorter duration, higher intensity exercise (for example, approximately 1 h at 75 % of maximal oxygen uptake; *V* O_2max_). The mechanism behind these performance improvements is completely different. In fact, it was demonstrated that when glucose was infused into the systemic circulation, this glucose was taken up at high rates but no performance effect was found [16]. This provides evidence that increasing glucose availability, as a substrate to the working muscle, has no effect during this type of activity. Interestingly, however, when individuals rinsed their mouths with a carbohydrate solution it resulted in performance improvements [11] that were very similar to the improvements seen with carbohydrate ingestion. There are now numerous studies that, on balance, demonstrate that this effect is real. Those studies are reviewed in several recent papers [10–14]. This would suggest that the beneficial effects of carbohydrate feeding during exercise are not confined to its conventional metabolic advantage, but may also contribute to a more positive afferent signal capable of modifying motor output [17]. These effects are specific to carbohydrates and are independent of taste [18].

It is known that whenever food or drink is placed in the mouth, taste receptor cells are stimulated and provide the first analysis of potentially ingestible food [19–21]. Taste receptor cells exist in groups of 50–100 in the taste buds, which are distributed across different papillae of the tongue, soft palate, and epiglottis [22]. Electrical activity initiated by a taste cue is transmitted to gustatory neurons (cranial nerves VII, IX, and X) that innervate the taste buds [23, 24]. This information converges on the nucleus of the solitary tract in the medulla, and is subsequently relayed by the ventral posterior medial nucleus of the thalamus to the primary taste cortex, located in the anterior insula and adjoining frontal operculum, and the putative secondary taste cortex located in the orbitofrontal cortex [19]. The primary taste cortex and orbitofrontal cortex have projections to regions of the brain, such as the dorsolateral prefrontal cortex, anterior cingulate cortex, and ventral striatum, which are thought to provide the link between gustatory pathways and the appropriate emotional, cognitive, and behavioral response [25, 26]. The fact that many of these higher brain regions have been reported to be activated by oral carbohydrates and not non-nutritive sweeteners [18, 27, 28] may provide a mechanistic explanation for the positive effects of a carbohydrate mouth rinse on exercise performance.

However, the receptors in the oral cavity that mediate these effects relating to performance have not yet been identified, and the exact roles of the various brain areas are not clearly understood. The taste receptor cells that are involved are not actually detecting taste but rather carbohydrate or energy.

**Fig. 1:**
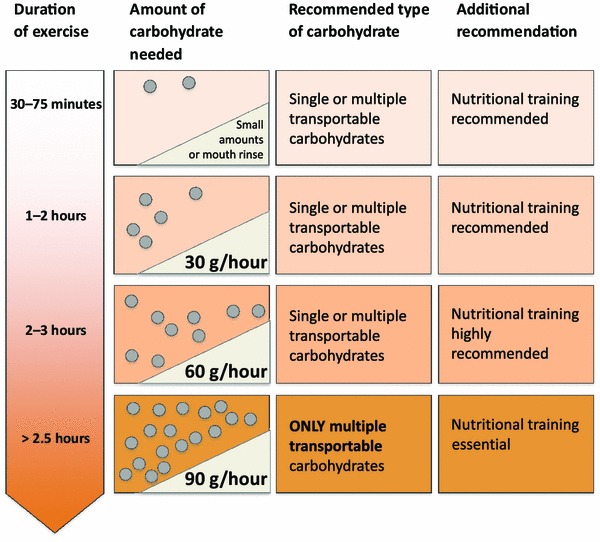
The new carbohydrate intake guidelines. Carbohydrate intake recommendations during exercise depend on the duration of exercise. In general, carbohydrate intake recommendations increase with increasing duration. The type of carbohydrate may also vary as well as recommendations for nutritional training. These recommendations are for well trained athletes. Aspiring athletes may need to adjust these recommendations downwards

Further research is warranted to understand fully the separate taste transduction pathways for various types of carbohydrates and how these differ between mammalian species, particularly in humans. However, it has been convincingly demonstrated that carbohydrate is detected in the oral cavity by unidentified receptors, and that this can be linked to improvements in exercise performance (for a review see Jeukendrup and Chambers [11]). The new guidelines suggested here take these findings into account (Fig. 1).

### Practical Implications of the Mouth Rinse Studies

These results suggest that it is not necessary to ingest large amounts of carbohydrate during exercise lasting approximately 30 min to 1 h and that a mouth rinse with carbohydrate may be sufficient to obtain a performance benefit (Fig. 1). In most conditions, the performance effects with the mouth rinse were similar to ingesting the carbohydrate drink, so there does not seem to be a disadvantage of consuming the drink, although occasionally athletes may complain of gastrointestinal distress when consuming larger amounts. When the exercise is more prolonged (2 h or more), carbohydrate becomes a very important fuel, and to prevent a decrease in performance it is essential to ingest carbohydrate. As discussed in the following two sections, larger amounts of carbohydrate may be required for more prolonged exercise.

## Prolonged Exercise and Multiple Transportable Carbohydrates

Different carbohydrates ingested during exercise may be used at different rates [12], but until a landmark publication in 2004 [29] it was believed that carbohydrate ingested during exercise could only be oxidized at a rate no higher than 1 g/min (60 g/h) independent of the type of carbohydrate [9]. This is reflected in guidelines published by the ACSM, which recommend that athletes should take between 30 and 60 g of carbohydrate during endurance exercise (over 1 h) [30] or 0.7 g/kg per hour [6].

It appears that exogenous carbohydrate oxidation is limited by the intestinal absorption of carbohydrates. It is believed that glucose uses the sodium-dependent transporter (SGLT1) for absorption, which becomes saturated at a carbohydrate intake of around 60 g/h. When glucose was ingested at this rate, and another carbohydrate (fructose) that uses a different transporter was ingested simultaneously, oxidation rates were well above 1 g/min (1.26 g/min) [29]. A series of studies followed in an attempt to work out the maximal rate of exogenous carbohydrate oxidation. In those studies, the rate of carbohydrate ingestion was varied and the types and combinations of carbohydrates varied. All studies confirmed that multiple transportable carbohydrates resulted in (up to 75 %) higher oxidation rates than carbohydrates that use SGLT1 only (for reviews see Jeukendrup [12, 15]). Interestingly, such high oxidation rates could not only be achieved with carbohydrate ingested in a beverage but also as a gel [31] or a low-fat, low-protein, low-fiber energy bar [32].

There are several studies that link the increased exogenous carbohydrate oxidation rates observed with multiple transportable carbohydrates to delayed fatigue and improved exercise performance. In one study, subjects ingested 1.5 g/min of glucose:fructose or glucose during 5 h of moderate-intensity exercise, and it was observed that the subjects’ ratings of perceived exertion were lower with the mixture of glucose and fructose than with glucose alone. Cyclists were also better able to maintain their cadence towards the end of 5 h of cycling [33]. Rowlands et al. [34] confirmed these findings and reported reduced fatigue when ingesting a maltodextrin:fructose mix (maltodextrin is a glucose polymer with little sweetness that is very rapidly digested and therefore behaves identical to glucose). It was also demonstrated that a glucose:fructose drink could improve exercise performance [35]. Cyclists exercised for 2 h on a cycle ergometer at 54 % *V*O_2max_ during which they ingested either a carbohydrate drink or placebo, and were then asked to perform a time trial that lasted approximately another 60 min. When the subjects ingested a glucose drink (at 1.8 g/min), they improved their power output by 9 % (254 vs. 231 W). However, when they ingested a glucose:fructose drink, there was another 8 % improvement of the power output over and above the improvement by glucose ingestion (275 vs. 254 W). This was the first study to show that exogenous carbohydrate oxidation rates may be linked to performance and the first to demonstrate a clear performance benefit with glucose:fructose compared with glucose [35]. These findings were reproduced by Triplett et al. [36] who found very similar performance improvements with glucose:fructose over glucose alone.

Rowlands et al. [37] recently took the research one step further and studied trained cyclists in mountain bike races (average 141 min) and laboratory trials (94-min high-intensity intervals followed by 10 maximal sprints). Carbohydrate solutions (maltodextrin:fructose or glucose:fructose in 2:1 ratios) were ingested at an average rate of 1.2 g carbohydrate/kg per hour (or 95 g/h). The maltodextrin:fructose solution substantially reduced race time by 1.8 % and abdominal cramps by 8.1 points on a 0–100 scale. After accounting for gastrointestinal discomfort, the effect of the maltodextrin:fructose solution on lap time was reduced by 1.1 %, suggesting that gastrointestinal discomfort explained part of the effect of maltodextrin:fructose on performance. In the laboratory, mean sprint power was enhanced by 1.4 % with fructose:maltodextrin.

Performance benefits have generally been observed in studies that are 2.5 h or longer and effects start to become visible in the third hour of exercise [33]. When exercise duration is shorter, multiple transportable carbohydrates may not have the same performance benefits [38], but it must be noted that the effects are at least similar to other carbohydrate sources.

## Carbohydrate during Exercise and Performance: Dose Response

Very few, well controlled, dose–response studies on carbohydrate ingestion during exercise and exercise performance have been published. Most of the older studies had serious methodological issues that made it difficult to establish a true dose–response relationship between the amount of carbohydrate ingested and performance. Until a few years ago, the conclusion seemed to be that a minimum amount of carbohydrate was needed (probably ~20 g/h based on one study) but it was generally assumed that there was no dose–response relationship [6].

More recently, however, evidence has been accumulating for a dose–response relationship between carbohydrate ingestion rates, exogenous carbohydrate oxidation rates, and performance. In one recent carefully conducted study, endurance performance and fuel selection was measured during prolonged exercise while ingesting glucose (15, 30, and 60 g/h) [39]. Twelve subjects cycled for 2 h at 77 % of peak oxygen uptake followed by a 20-km time trial. The results suggest a relationship between the dose of glucose ingested and improvements in endurance performance. The exogenous glucose oxidation increased with ingestion rate and it is possible that an increase in exogenous carbohydrate oxidation is directly linked with, or responsible for, exercise performance.

A large-scale multicenter study by Smith et al. [40] also investigated the relationship between carbohydrate ingestion rate and cycling time trial performance to identify a range of carbohydrate ingestion rates that would enhance performance. In their study, across four research sites, 51 cyclists and tri-athletes completed four exercise sessions consisting of a 2-h constant load ride at a moderate to high intensity. Twelve different beverages (consisting of glucose:fructose in a 2:1 ratio) were compared, providing participants with 12 different carbohydrate doses in the range of 10–120 g carbohydrate per hour during the constant load ride. The carbohydrates used were multiple transportable carbohydrates (glucose:fructose). At all four sites, a common placebo was provided that was artificially sweetened, colored, and flavored and did not contain carbohydrate. The order of the beverage treatments was randomly assigned at each site (three at each site). Immediately following the constant load ride, participants completed a computer-simulated 20-km time trial as quickly as possible. The ingestion of carbohydrate significantly improved performance in a dose-dependent manner and the authors concluded that the greatest performance enhancement was seen at an ingestion rate between 60 and 80 g of carbohydrate per hour. Interestingly, these results are in line with an optimal carbohydrate intake proposed by a recent meta-analysis [41]. Based on the studies mentioned above, new carbohydrate intake recommendations for more prolonged exercise can be formulated and are listed in Fig. 1 and Sect. 5.

## Recommendations for Carbohydrate Intake During Different Endurance Events

- Recommendations for carbohydrate intake during exercise (see Fig. 1) are dependent on exercise duration, the absolute exercise intensity, as well as the sport and its rules and regulations.
- Athletes who perform at absolute intensities that are lower will have lower carbohydrate oxidation rates and the amounts presented in Fig. 1 should be adjusted (downwards) accordingly.
- The recommended carbohydrate intake can be achieved by consuming drinks, gels, or low-fat, low-protein, and low-fiber solid foods (bars), and selection should be based on personal preference.
- Athletes can adopt a mix-and-match strategy to achieve their carbohydrate intake goals.
- Carbohydrate intake should be balanced with a fluid intake plan based on fluid needs, and it must be noted that solid foods and highly concentrated carbohydrate solutions have been shown to reduce fluid absorption.
- It is highly recommended to train/practise the nutrition strategy for competition to reduce the chances of gastrointestinal discomfort and to increase the absorptive capacity of the intestine.

## Training Status

A question that often arises is whether the results of those studies (often conducted in trained or even very well trained individuals) may translate to less trained or untrained individuals. A few studies compared a group of trained individuals with untrained individuals. For example, we compared substrate use in trained and untrained men during exercise with glucose ingestion [42]. All men exercised at approximately 60 % of their *V* O_2max_, with the trained men exercising at a significantly higher absolute exercise intensity. Glucose was ingested at regular intervals and the average intake was approximately 1.1 g/min. Total carbohydrate oxidation was similar in both groups, but fat oxidation and energy expenditure were higher in the trained men. Interestingly, even though the trained men were exercising at a 40 % higher absolute power output, exogenous glucose oxidation was not different between the two groups (0.95 g/min in trained men and 0.96 g/min in untrained men) [42]. In a follow-up study, trained and untrained subjects were studied at the same relative but also absolute exercise intensity [43]. Again, no differences were found in exogenous carbohydrate oxidation between trained and untrained subjects as exogenous carbohydrate oxidation was similar in all trials. Even the trained subjects, who exercised at two different intensities, showed no difference in exogenous carbohydrate oxidation between these intensities [43].

It must be noted, however, that the untrained subjects in both of those studies [42, 43] had *V* O_2max_ values that are higher than the sedentary population, so guidelines may be extrapolated to athletes of different levels, but not necessarily to the sedentary population. However, in the study by van Loon et al. [43] in which the absolute exercise intensity did not make a difference, it is possible that there is a threshold below which exogenous oxidation rates are lower, and all subjects in those studies always exercised above that absolute intensity threshold.

Perhaps it is not the training status of the athlete that is important but the absolute exercise intensity and the absolute rates of carbohydrate oxidation that determines exogenous carbohydrate oxidation rates. It is unlikely that the runner who completes the marathon in 5 h would not necessarily need an intake of 90 g of carbohydrate per hour as this would be close to, or could even exceed, the total carbohydrate use at that absolute exercise intensity.

## The Effect of Exercise Intensity

Carbohydrate needs may be different at different exercise intensities. When the exercise intensity is low and total carbohydrate oxidation rates are low, carbohydrate intake recommendations may have to be adjusted downwards. There are actually surprisingly few studies to base firm recommendations on. With increasing exercise intensity, the active muscle mass becomes more and more dependent on carbohydrate as a source of energy. Both an increased muscle glycogenolysis and increased plasma glucose oxidation will contribute to the increased energy demands [44]. It is therefore reasonable to expect that exogenous carbohydrate oxidation will increase with increasing exercise intensities. Indeed, an early study by Pirnay et al. [45] reported lower exogenous carbohydrate oxidation rates at low exercise intensities compared with moderate intensities, but exogenous carbohydrate oxidation tended to level off between 51 and 64 % *V* O_2max_. There was no difference in exogenous carbohydrate oxidation between 60 and 75% *V* O_2max_ [45].

It is therefore possible that lower exogenous carbohydrate oxidation rates are only observed at very low exercise intensities when the reliance on carbohydrate as an energy source is minimal. In this situation, part of the ingested carbohydrate may be directed towards nonoxidative glucose disposal (storage in the liver or muscle) rather than towards oxidation.

## Effect of Body Weight

The guidelines for carbohydrate intake during exercise, presented here, are expressed in grams per hour of exercise and these figures are not corrected for body weight (BW). In the most recent position statement by the American Dietetics Association and the ACSM [6], advice with respect to carbohydrate intake during exercise is expressed in grams per kilogram. The rationale for this is unclear as there appears to be no correlation between BW and exogenous carbohydrate oxidation [12]. The reason for this lack of correlation between BW and exogenous carbohydrate oxidation is probably that the limiting factor is carbohydrate absorption and absorption is largely independent of BW. It is likely, however, that the absorptive capacity of the intestine is modified by the carbohydrate content of the diet, as it has been shown in animal studies that intestinal transporters can be upregulated with increased carbohydrate intake. As exogenous carbohydrate is independent of BW or muscle mass, but dependent on absorption and to some degree the absolute exercise intensity (at very low absolute intensities, low carbohydrate rates may also restrict exogenous carbohydrate oxidation), the advice given to athletes should be in absolute amounts. These results clearly show that there is no rationale for expressing carbohydrate recommendations for athletes per kilogram of BW (Sect. 5).

In summary, individual differences in exogenous carbohydrate oxidation exist, although they are generally small. These differences are not related to BW but more likely to a capacity to absorb carbohydrates. This in turn could be diet related.

## Training the Gut

As the absorption of carbohydrate limits exogenous carbohydrate oxidation, and exogenous carbohydrate oxidation seems to be linked with exercise performance, an obvious potential strategy would be to increase the absorptive capacity of the gut. Anecdotal evidence in athletes suggests that the gut is trainable and that individuals who regularly consume carbohydrate or have a high daily carbohydrate intake may also have an increased capacity to absorb it. Intestinal carbohydrate transporters can indeed be upregulated by exposing an animal to a high-carbohydrate diet [46]. To date there is limited evidence in humans. A recent study by Cox et al. [47] investigated whether altering daily carbohydrate intake affects substrate oxidation and in particular exogenous carbohydrate oxidation. It was demonstrated that exogenous carbohydrate oxidation rates were higher after the high-carbohydrate diet (6.5 g/kg BW/day; 1.5 g/kg BW provided mainly as a carbohydrate supplement during training) for 28 days compared with a control diet (5 g/kg BW/day). This study provided evidence that the gut is indeed adaptable and this can be used as a practical method to increase exogenous carbohydrate oxidation. We recently suggested that this may be highly relevant to the endurance athlete and may be a prerequisite for the first person to break the 2-h marathon barrier [48]. Although more research is needed, it is recommended to practise the carbohydrate intake strategy in training, and dedicate at least some time to training with a relatively high carbohydrate intake.

## Carbohydrate Intake in Real-Life Events

Relatively few studies have investigated how much carbohydrate athletes ingest during races and whether they meet the recommended guidelines. In a study by Kimber et al. [49], the average carbohydrate intake during an ironman distance triathlon was 1.0 g/kg BW/h in female tri-athletes and 1.1 g/kg BW/h in male tri-athletes. They achieved these carbohydrate intakes by ingesting very large amounts of carbohydrate during cycling (approximately 1.5 g/kg BW/h). Most of the intake occurred during the cycling leg in which intake was almost three times as high as during the running leg. In male athletes, carbohydrate intake was positively correlated with finish time but this relationship was not confirmed in women. A large study of endurance events by Pfeiffer et al. [50] demonstrated a wide variation in carbohydrate intake reported by athletes between and within events, with the highest intakes in cycling and triathlon events and the lowest in marathons. In that study, it was also found that in ironman races, carbohydrate intake was related to finish time, with greater carbohydrate intake correlating to better performance. These findings appear to be in agreement with the recent dose–response studies by Smith and colleagues [39, 51].

## Different Advice for Different Endurance Sports

With carbohydrate feeding during cycling it has repeatedly been shown that muscle glycogen breakdown is unaffected. During running, however, there are suggestions that muscle glycogen breakdown is reduced in particular in type I muscle fibers [52]. Therefore, carbohydrate feeding results in improved performance in cycling and running, although the mechanism by which this occurs may not necessarily be the same. This issue is discussed in more detail in an excellent review by Tsintzas and Williams [53]. Exogenous carbohydrate oxidation appears to be similar in cycling and running [54], suggesting that the advice for cyclists and runners is not different.

### Intermittent and Skill Sports

The vast majority of studies have been performed with endurance athletes performing continuous exercise. Most team sports have a highly intermittent nature, with bursts of very high intensity exercise followed by relatively low intensity recovery periods. Furthermore, performance in these sports is often dependent on other factors than the maintenance of speed or power, and factors such as agility, timing, motor skill, decision making, jumping, and sprinting may all play a role. Nevertheless, carbohydrate ingestion during exercise has also been shown to enhance endurance capacity in intermittent activities. A large number of studies have demonstrated that if carbohydrate is ingested during intermittent running, fatigue can be delayed and time to exhaustion can be increased [55–59].

More recently, studies have incorporated measurements of skill into their performance measurements. Currell et al. [60] developed a 90-min soccer simulation protocol that included measurements of skill, such as agility, dribbling, shooting, and heading. The soccer players performed 90 min of intermittent exercise that mimicked their movement patterns during a game. During the 90 min, skill performance measurements were performed at regular intervals. Agility, dribbling, and accuracy of shooting were all improved but heading was not affected with carbohydrate ingestion. Other studies have found similar effects [61]. Although typically some of the skills measured in these studies were improved with carbohydrate feeding, the mechanisms behind these improvements are unknown and have not been studied in any detail.

It appears that carbohydrate intake during team sports, and other sports with an element of skill, has the potential to improve not only fatigue resistance but also the skill components of a sport, especially towards the end of a game. The practical challenge is often to find ways to ingest carbohydrate during a game within the rules of the sport.

## Conclusion

In summary, there have been significant changes in the understanding of the role of carbohydrates during exercise in recent years, and this allows for more specific and more individualized advice with regard to carbohydrate ingestion during exercise. The new proposed guidelines take into account the duration (and intensity) of exercise, the advice is not restricted to the amount of carbohydrate, and they also give direction with respect to the type of carbohydrate. The recommendations presented here are derived mostly from studies with trained and well trained athletes. Athletes who perform at lower absolute intensities will have lower carbohydrate oxidation rates, and the amounts presented here should be adjusted (downwards) accordingly. The recommended carbohydrate intake can be achieved by consuming drinks, gels, or low fat, low-protein, and low-fiber solid foods (bars), and selection should be determined by personal preference. Athletes can adopt a mix-and-match strategy to achieve their carbohydrate intake goals. However, the carbohydrate intake should be balanced with a fluid intake plan, and it must be noted that solid foods and highly concentrated carbohydrate solutions have been shown to reduce fluid absorption. Although a slowing of gastric emptying and absorption can be partly prevented by using multiple transportable carbohydrates, this is something the athlete needs to consider when developing his or her nutrition strategy. Although more research is needed, it is highly recommended that athletes test their nutrition strategy in training to reduce the chances of gastrointestinal discomfort and to increase the absorptive capacity of the intestine.

Finally, it must be noted that most studies are based on findings in runners and cyclists, and more work is needed to establish the effects and underlying mechanisms of carbohydrate ingestion on skill components in intermittent team sports. Recommendations have been summarized in Fig. 1 and Sect. 5.
